# Critical issue on the *extinction and inattention* subtest of NIHSS scale: an analysis on post-acute stroke patients attending inpatient rehabilitation

**DOI:** 10.1186/s12883-021-02499-9

**Published:** 2021-12-08

**Authors:** Benedetta Basagni, Bahia Hakiki, Silvia Campagnini, Emilia Salvadori, Antonello Grippo, Anita Paperini, Chiara Castagnoli, Ines Hochleitner, Angela Maria Politi, Paola Gemignani, Irene Eleonora Mosca, Azzurra Franceschini, Enrico Bacci Bonotti, Alessandro Sodero, Andrea Mannini, Leonardo Pellicciari, Anna Poggesi, Claudio Macchi, Maria Chiara Carrozza, Francesca Cecchi

**Affiliations:** 1 IRCCS Fondazione Don Carlo Gnocchi, Via di Scandicci 269 -, 50143 Florence, Italy; 2 The Biorobotics Institute, Scuola Superiore Sant’Anna, Pontedera, Italy; 3 Department of Experimental and Clinical Medicine, University of Florence, Florence, Italy; 4 Department of Neuroscience, Psychology, Drug Research and Child Health, University of Florence, Florence, Italy

**Keywords:** Stroke, NIHSS, Hemineglect, Heminattention, Visual field, Rehabilitation

## Abstract

**Objectives:**

This study aims to evaluate the diagnostic performance of NIHSS *extinction and inattention* item, compared to the results of the Oxford Cognitive Screen (OCS) heart subtest. Additionally, the possible role of the NIHSS *visual field* subtest on the NIHSS *extinction and inattention* subtest performance is explored and discussed.

**Methods:**

We analysed scores on NIHSS *extinction and inattention* subtest, NIHSS *visual field* subtest, and OCS *heart subtest* on a sample of 118 post-stroke patients.

**Results:**

Compared to OCS heart subtest, the results on NIHSS *extinction and inattention* subtest showed an accuracy of 72.9% and a moderate agreement level (Cohen’s kappa = 0.404). Furthermore, a decrease in NIHSS accuracy detecting neglect (61.1%) was observed in patients with pathological scores in NIHSS *visual field* item.

**Conclusions:**

Extreme caution is recommended for the diagnostic performance of *extinction and inattention* item of NIHSS. Signs of neglect may not be detected by NIHSS, and may be confused with visual field impairment.

**Trial registration:**

This study refers to an observational study protocol submitted to ClinicalTrials.gov with identifier: NCT03968627. The name of the registry is “Development of a National Protocol for Stroke Rehabilitation in a Multicenter Italian Institution” and the date of the registration is the 30th May 2019.

## Introduction

The National Institutes of Health Stroke Scale (NIHSS) is widely used to assess the severity of acute stroke [1, 2]. The NIHSS is an 11-item test assessing the main domains of stroke related disability: level of consciousness, gaze anomalies, visual field restriction, facial palsy, motor arm and leg limitations, limb ataxia, sensory deficits, aphasia, dysarthria, and extinction and inattention (formerly called neglect). Each subtest variably scores between 0 and 4 or less and the total score of NIHSS is obtained by their sum.

Given a maximum total score of 42, a score of 0 represents normal function, whereas higher scores indicate more severe degrees of impairment. More in detail, scores between 1 and 4 indicate a mild stroke, scores between 5 and 14 indicate a mild to moderate stroke, scores between 15 and 24 indicate moderate to severe stroke, whereas scores between 25 and 42 indicate very severe stroke [1].

Since its first publication, NIHSS scale has had a great spread both in clinical practice and research trials [3]. It is considered a valid tool to determine the impairment and to predict outcome in patients affected by stroke [4].

NIHSS scale has the undoubtable advantage of allowing a quick and reliable assessment of patients’ deficits and it is crucial in the acute phase to facilitate decision-making on thrombolysis in patients with ischemic stroke [5]; it is also extremely useful in supporting a standardised communication among clinicians [6].

Nevertheless, similarly to other global and observational screens, it has some limitations. Previous works highlighted that NIHSS can underrepresent both posterior circulation and right hemisphere lesions [7, 8]. In particular, with respect to cognitive impairments, several Authors demonstrated that NIHSS could be not sensitive enough [9]. Gottesman et al. [7] underlined its susceptibility to floor effects and the trend to bias towards hemisphere-specific lesions. Abzhandadze et al. [10] compared the cognitive subscale of the NIHSS with a reference standard neuropsychological test: results on 531 patients showed that NIHSS had a limited ability to identify cognitive deficits in acute stroke.

More specifically, Moore et al. [11] focussed on NIHSS *extinction and inattention subtest*, the item supposed to detect hemineglect. Hemineglect is a pathological condition characterised by reduced awareness of stimuli on one side of space, in the absence of sensory loss. It usually concerns left hemispace as a consequence of right hemisphere lesions, but it is sometimes present after left hemisphere lesions affecting right hemispace. According to the spatial domains, neglect can be divided into *personal* (referred to body), *peripersonal* (within arm’s reach) and *extrapersonal* (beyond arms reach). Previous research revealed that the severity of unilateral spatial neglect in acute stroke is negatively associated to the degree of long-term disability and functional independence [12, 13]; for this reason, neglect early detection is highly relevant for the formulation of individualised rehabilitation programs and the identification of final functional outcomes.

Moore et al. [11] demonstrated a poor sensitivity of the NIHSS *extinction and inattention subtest*, compared with a cancellation task (heart task of the Oxford Cognitive Screen, OCS). The authors concluded that NIHSS alone is not enough to detect heminattention symptoms and that at least a simple cancellation task is needed for a reliable detection of post-stroke neglect. Interestingly, they suggested that the frequent failure of neglect diagnosis, based on clinical observation exclusively, may be influenced by visual field deficits (e.g., hemianopia). Indeed, the double simultaneous stimulation that allows to assign a score to the NIHSS *extinction and inattention* subtest is performed at the end of the visual field examination, augmenting the possibility to confuse the disturbances, especially when co-occurring (see Table 1 for item 3 and 11 NIHSS instructions).

**Table 1:** NIHSS instructions

***Item 3.*** Visual fields (upper and lower quadrants) are tested by confrontation, using finger counting or visual threat, as appropriate. Patients may be encouraged, but if they look at the side of the moving fingers appropriately, this can be scored as normal. If there is unilateral blindness or enucleation, visual fields in the remaining eye are scored. Score 1 only if a clear-cut asymmetry, including quadrantanopia, is found. If patient is blind from any cause, score 3. Double simultaneous stimulation is performed at this point. If there is extinction, patient receives a 1, and the results are used to respond to item 11.	**0** = No visual loss. **1** = Partial hemianopia. **2** = Complete hemianopia. **3** = Bilateral hemianopia (blind including cortical blindness).
***Item 11.*** Extinction and Inattention (formerly Neglect): Sufficient information to identify neglect may be obtained during the prior testing. If the patient has a severe visual loss preventing visual double simultaneous stimulation, and the cutaneous stimuli are normal, the score is normal. If the patient has aphasia but does appear to attend to both sides, the score is normal. The presence of visual spatial neglect or anosognosia may also be taken as evidence of abnormality. Since the abnormality is scored only if present, the item is never untestable.	**0** = No abnormality. **1** = Visual, tactile, auditory, spatial, or personal inattention or extinction to bilateral simultaneous stimulation in one of the sensory modalities. **2** = Profound hemi-inattention or extinction to more than one modality; does not recognize own hand or orients to only one side of space.

The present work aims to study the diagnostic performance of the NIHSS *extinction and inattention* subtest (Neis), administered to a cohort of stroke inpatients attending post-acute intensive rehabilitation, compared to heart task of the OCS test [14, 15], a cancellation test considered to be highly accurate to detect peripersonal neglect [16]. The purpose of this study was primarily to replicate the results of Moore et al. [11] on Neis for post-stroke patients, and additionally to explore the role of the *visual field* subtest (Nvfs) on the Neis diagnostic performance.

## Methods

### Participants and experimental setting

This work used data from a large prospective multicentric study aiming at the identification of potential predictors of functional recovery in post-acute stroke patients attending intensive inpatient rehabilitation in Fondazione Don Carlo Gnocchi (RIPS study) [17].

The protocol required the systematic inclusion of patients attending post-acute stroke intensive rehabilitation according to an integrated care pathway [18], with the following inclusion criteria: a) first-ever or recurrent ischemic or haemorrhagic stroke; b) stroke diagnosis confirmed clinically and by brain imaging; c) acute event within 30 days; d) age 18+; e) written informed consent. Patients with transitory ischemic attack or severe acquired brain injury, according to the Italian Guidelines of Rehabilitation published in 1998 [19], were excluded. Further details on the study protocol were described elsewhere [17].

Of the 241 patients recruited in RIPS study, for this analysis we included only patients for whom Neis and Nvfs scores, together with the Oxford Cognitive Screen (OCS), were available. The presence of some clinical conditions, as well as severe sensory/motor deficits, did not allow data collection in all subjects. The patients underwent the neurological and neuropsychological assessment in the first week from the admission in the rehabilitation centre, immediately after discharge from the acute hospital unit.

### Evaluation tools

The full NIHSS was administered by a certified neurologist or physiatrist medical doctor (http://nihss-neurosapienza.trainingcampus.net). In this study, only the two subtest of *visual field* and *extinction and inattention* were considered for analysis:

- NIHSS *visual field* (Nvfs): According to NIHSS original instructions, visual fields are tested by confrontation, using finger counting or visual threat in upper and lower quadrants. The score is 0 if no visual loss is revealed, 1 for quadrantanopia, 2 for complete and 3 for bilateral hemianopia (blindness, including cortical one). Double simultaneous stimulation is also performed during this examination. If there is extinction, patient receives a 1, and the results are used to respond to the item *extinction and inattention*.
- NIHSS *extinction and inattention* (Neis): This is the last subtest proposed during NIHSS administration. Following instruction, sufficient information to identify neglect should be obtained during the prior testing. Scoring includes point 0 for no abnormality, 1 for visual, tactile, auditory, spatial, or personal inattention or extinction to bilateral simultaneous stimulation in one of the sensory modalities, and 2 for profound heminattention or extinction to more than one modality. The instructions do not further specify which tasks should guide the examiner towards neglect signals, beyond double stimulation.

The Oxford Cognitive Screen is a short cognitive screening tool for stroke evaluation of attention, language, praxis, number and memory. The full scale was administered by a neuropsychologist. Only the subtest of hemineglect (heart test) was considered in this study:

- OCS heart test (Ohs) is a cancellation task in which outline drawings of 150 heart are shown pseudo-randomly scattered over an A4 sheet. One-third of the heart are complete (50 targets) and two-thirds are open, either on the left or the right-hand side (distractors). Targets and distractors are evenly distributed. Three indexes can be obtained: a total score, and two asymmetry scores. The total score represents the total number of complete hearts cancelled within a limited time. Instead, asymmetries scores represent difference between complete hearts cancelled in a specific portion of the page (space asymmetry, as a sign of egocentric neglect), and difference between left- and right-broken hearts (object asymmetry as suggestive of allocentric neglect). Space asymmetry score is obtained through the difference between the targets found in the right and left half-sheet. A positive total score indicates that the patient cancelled more right-side hearts than left-side and it is showing left egocentric neglect (neglect of the left side of space). Conversely, a negative score indicates that more left-side than right-side hearts were cancelled and the patient is showing right egocentric neglect. The test also enables to detect the presence of allocentric neglect through “object asymmetry score”, but since NIHSS does not allow to identify this characteristic of neglect, this data was not considered in this work.

### Statistical analysis

Statistical analyses were conducted on IBM SPSS Statistics for Windows, Version 26.0. Armonk, NY: IBM Corp.

For the primary endpoint, the diagnostic performance of the Neis was investigated through the calculation of sensitivity, specificity and accuracy values, compared to the pencil-paper heart test of the OCS. For this analysis, the dichotomised versions of the variables were obtained considering for the Neis a score of 0 as absence of neglect and 1 or greater as presence of neglect, whilst for the Ohs a score in absolute values at space asymmetry score greater than 2 as presence of neglect and absence elsewhere [15]. In order to investigate the influence of the visual field performances on the neglect assessment, the same analysis was repeated on the subgroups of patients with normal and pathological visual field, according to Nvfs. Additionally, to evaluate the association with the scores severity, a contingency table was created using the Neis raw scores against the dichotomised Ohs. Sensitivity, specificity and accuracy were obtained. The Cohen’s kappa test for the agreement rate among the two evaluation tools was also performed.

## Results

Of the 241 patients recruited, the analysis involved a total of 118 patients. The main characteristics of the subjects are represented in Table 2.

**Table 2:** Population characteristics

***Variable***	***Mean (std) / Median [IQR] / Frequencies***
Age (years)	77 [16]
Gender (M: Male; F: Female)	M: 64; F: 54
Centre (Fi: Firenze; Ma: Massa; Fv: Fivizzano; Sp: La Spezia)	Fi: 75; Ma: 10; Fv: 5; Sp: 28
Stroke type (1: Ischemic; 2: Haemorrhagic)	1: 88; 2: 30
Lesion side (1: Right; 2: Left; 3: Bilateral)	1: 58; 2: 47; 3: 9
Time from event (days)	10.5 [9]

For what concerns the evaluation tools considered within the study, 24 patients over 118 resulted diagnosed with neglect on the Neis, whilst 50 patients out of 118 resulted with an altered score on the Ohs (Table 3).

**Table 3:** Contingency tables for Neis with respect to the Ohs considering the whole population (top), only the subjects with normal (middle) and altered (bottom) visual field respectively. The tables report respectively the relative frequency in number and as percentage with respect to the total of Neis, with respect to the total of Ohs and with respect to the total of participants

		***Ohs***		
		0	1	Total
a) All subjects				
***Neis***	0	65	29	94
		69.1%	30.9%	100%
		95.6%	58%	79.7%
		55.1%	24.6%	79.7%
	1	3	21	24
		12.5%	87.5%	100%
		4.4%	42%	20.3%
		2.5%	17.8%	20.3%
	Total	68	50	118
		57.6%	42.4%	100%
		100%	100%	100%
		57.6%	42.4%	100%
b) Subjects with normal visual field				
***Neis***	0	64	24	88
		72.7%	27.3%	100%
		98.5%	68.6%	88%
		64%	24%	88%
	1	1	11	12
		8.3%	91.7%	100%
		1.5%	31.4%	12%
		1%	11%	12%
	Total	65	35	100
		65%	35%	100%
		100%	100%	100%
		65%	35%	100%
c) Subjects with altered visual field				
***Neis***	0	1	5	6
		16.7%	83.3%	100%
		33.3%	33.3%	33.3%
		5.6%	27.8%	33.3%
	1	2	10	12
		16.7%	83.3%	100%
		66.7%	66.7%	66.7%
		11.1%	55.6%	66.7%
	Total	3	15	18
		16.7%	83.3%	100%
		100%	100%	100%
		16.7%	83.3%	100%

First, the sensitivity, specificity and accuracy of the Neis with respect to Ohs were calculated. Compared to the Ohs reference, the NIHSS obtained a low sensitivity (56.8%), a higher specificity (95.6%), and an accuracy of 72.9%.

The influence of visual field alterations on the NIHSS evaluation of the neglect was analysed comparing sensitivity, specificity and accuracy values on the subgroups obtained by altered and normal results on the Nvfs test. A normal visual field was considered for subjects with a raw score of 0 and altered elsewhere, in NIHSS visual field item. The subgroup of patients with altered visual field score, showed a smaller accuracy with respect to the other group (61.1 and 75.0% respectively) (Table 3).

**Fig. 1:**
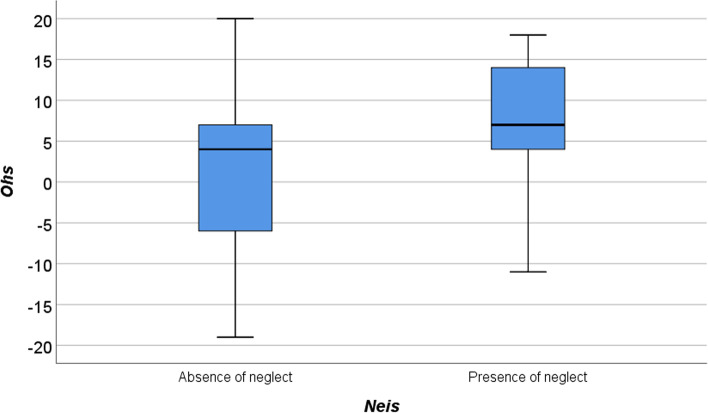
Boxplot representing the Ohs raw scores for the 50 patients with diagnosed neglect on the Ohs. It is presented the Ohs raw score separately for the 29 patients misclassified by Neis test (on the left) and the 21 patients correctly diagnosed with neglect (on the right)

Going more in detail, a contingency table was analysed using the raw score of the Neis with respect to the Ohs dichotomised. It emerged there is a good estimation of the pathological category with respect to the non-pathological one (Table 4), with a tendency in misclassification on those patients with more moderate levels of heminattention (Fig. 1).

**Table 4:** Contingency table between Neis raw score and Ohs dichotomised. The table reports respectively the relative frequency in number and as percentage with respect to the total of Neis, with respect to the total of Ohs and with respect to the total of participants

		***Ohs***		
		0	1	Total
***Neis_raw score***	0	65	29	94
		69.1%	30.9%	100%
		95.6%	58%	79.7%
		55.1%	24.6%	79.7%
	1	2	13	15
		13.3%	86.7%	100%
		2.9%	26%	12.7%
		1.7%	11%	12.7%
	2	1	8	9
		11.1%	88.9%	100%
		1.5%	16%	7.6%
		0.8%	6.8%	7.6%
	Total	68	50	118
		57.6%	42.4%	100%
		100%	100%	100%
		57.6%	42.4%	100%

Lastly, the agreement of the two measures was tested with the Cohen’s kappa, obtaining a k of 0.404 and corresponding to a moderate agreement level.

## Discussion

This study primarily aims to compare, on a sample of 118 post-acute stroke patients, the results on *extinction and inattention* NIHSS subtest with a more accurate test for peripersonal hemineglect (OCS heart subtest, Ohs). Additionally, this study investigates the influence of the *visual field* NIHSS subtest on the results of *extinction and inattention* item, given that hemianopsia and neglect frequently coexist and that the two disturbances may be confused in the stroke population [20].

Our results showed a poor sensitivity of NIHSS detecting peripersonal neglect (56.8%). Dichotomising the results (normal/pathological), Cohen’s kappa test between the two tests was moderate and the accuracy was 72.9%. Compared to the Ohs, NHISS *extinction and inattention subtest* had 29 false negatives, and 3 false positives. Therefore, in the 24.6% of cases, NIHSS was unable to detect neglect and, unexpectedly, in the 2.5% of cases NIHSS classified as inattention symptoms signs that were not detected in Ohs test.

Among the false negatives, NIHSS showed a tendency in misclassification especially on those patients with mild forms of heminattention, where, as it is intuitive, clinical observation may not be enough (Fig. 1). Nevertheless, some other considerations could be done. NIHSS may fail in detecting neglect, also because the scale considers extinction as a crucial aspect. The lack of extinction in visual or somatic bilateral stimulation, does not unequivocally mean that patients do not present hemineglect. Neglect is a heterogeneous syndrome and extinction is not the determining factor for the diagnosis. Although both neglect and extinction are typical syndromes of acute right hemispheric stroke and frequently co-occur, they do not overlap [21–23]. Hence, the principle used by the NIHSS scale to guide the clinician towards the diagnosis, may not be the most appropriate.

Regarding the false positives, one out of three presented a normal Nvfs. After the analysis of the patient’s performance, it was found in the clinical folder that he was affected by a selective extrapersonal neglect, and consequently correctly diagnosed with respect to hemianopia. In the other two cases, a confounding effect of visual field task may be supposed. Hemianopia and neglect frequently coexist, and a differential diagnosis is often difficult. Hemianopia may be misattributed to hemineglect and, equally, neglect impairments may be misattributed to hemianopia. In fact, patients with heminattention may fall into the manual visual field test, because they do not pay attention to information coming from the neglected side, even if they perceive it [24].

The results of the influence of the NIHSS *visual field subtest* on NIHSS accuracy in detecting neglect showed that when the Nvfs score resulted impaired, the accuracy of the NIHSS dramatically dropped (61.1%). Therefore, a visual field impairment could be in some cases confounded with neglect symptoms. Nevertheless, another issue has to be considered. In fact, three subtypes of neglect disturbances were aforementioned described: personal, peripersonal and extrapersonal. These subtypes may dissociate and considered to be related to region-specific lesions [25]. In the study of Spaccavento et al. [26] on 130 right stroke patients with neglect, 57% showed an overlap between two or three forms, 17% presented only the peripersonal form, 12% only the personal form and 11% only the extrapersonal form. In the absence of a clear definition of the exact distance in which NIHSS visual field task has to be performed, we cannot exclude an effect of a dissociation between peripersonal and extrapersonal neglect forms. Furthermore, the visual extinction test, that allows the clinician to score also the extinction and inattention test, is conducted at the end of the visual field examination, and therefore it could in the same way be affected by the bias described above.

Together with the probable influence of visual field subtest alterations, another aspect should also be considered. Indeed, in this study, we limited our analysis to the space asymmetry score, the measure on the heart test that is more informative concerning egocentric neglect [REF]. Nevertheless, the test includes two other measures (total omissions and object asymmetry scores), that allow for a more accurate interpretation of the results giving the information on the general attentional ability and the presence of allocentric neglect. However, by focussing solely on the latter two measures, misleading conclusions could be drafted concerning neglect diagnosis. In fact, a high number of total omissions could indicate both a very serious form of neglect (if concentrated in the omitted half-space), and more general attentional difficulties (if spread throughout the space). Similarly, a high object asymmetry score alone does not exclude the presence of additional global cognitive and attentive difficulties, besides the allocentric component of neglect. Therefore, we decided to focus on the asymmetry of space score exclusively, as a reliable measure of egocentric neglect, but we cannot exclude the presence in our sample of subjects without a “pure” syndromic pattern of neglect.

## Conclusions

From the results obtained in this study, extreme caution is recommended for conclusions over the presence of hemineglect outside the acute phase, from the only NIHSS *extinction and inattention* subtest, and over visual filed integrity from NIHSS *visual field* subtest. Some possible source of errors emerged from our analysis:

1. Patients may present hemineglect and not be diagnosed by NIHSS because they present mild form of neglect, or because they do not present visual or somatosensorial extinction.
2. The presence of visual field impairment, may induce clinicians to misattribution of such impairment to hemineglect.
3. Hemineglect may simulate hemianopia impairment.
4. Selective extrapersonal hemineglect impairment may be confounded with hemianopia.

## Data Availability

The data will be available at this link https://www.dropbox.com/sh/kwnljsg34la3ybc/AADpz0RF3CNo_XrI6eWwIP6ra?dl=0 upon acceptance and for research purposes upon request to the corresponding author (scampagnini@dongnocchi.it).
